# Francis Wilfred Willway

**Published:** 1944

**Authors:** 


### FRANCIS WILFRED WILLWAY, M.D., M.S. (London), F.R.C.S.

BRISTOL suffered a great loss in the death of Wilfred Willway on 6th January. He came to the Bristol Royal Infirmary in 1936 as Surgical Registrar, after a brilliant career at King's College Hospital. He was the son of the late Dr. Frederick Willway of Streatham, and more distantly traced back to the Olive family, well-known in Bristol. His special surgical study was Neuro-Surgery, and as Professor Geoffrey Jefferson wrote in the *B.M.J.* (January 29, 1944): "We Neuro-Surgeons cannot afford these losses from our ranks." Much of his operative Neuro-Surgery was carried out at the Burden Neurological

Institute. But his Casualty Surgery at the Bristol Royal Infirmary in the air raids of 1940-41 called for uncommon skill and made tremendous demands on his strength. For through all that time he was an ailing man.

He knew that his years would be short ; but he never flinched or stinted his efforts. To quote Jefferson again : " Few people realize that death is close upon them, and none that I have known have treated that knowledge with Willway's good-natured disdain."

Another colleague wrote of him in the *B.M.J.* :—

Wilfred Willway was one of the most brilliant students of King's College Hospital. He had that type of brain which makes its possessor able to play four games of chess and conduct a conversation at the same time. He never failed in any examination, took the F.R.C.S.Eng. and M.S.Lond. at an early age, and then the M.D. " just for fun," as he explained. He was a great teacher, whose students admired and loved him as did all his friends. As a man he was full of kindness and humility and yet charged with enthusiasm for his work and the welfare of his patients, students, and friends. Coming to Bristol from London, Willway entered fully into the teaching and professional work of the Bristol Hospitals and Medical School. He was a pillar of strength to the Hospitals' staff, who, because of his sound organizing powers, gave him charge of the war emergency arrangements at the Royal Infirmary. Willway was Bristol's first Neuro-Surgeon, and he was rapidly achieving a wide reputation for his work at the Infirmary and the Burden Neurological Institute. His courage was admirable, and in the course of a long day's operating it is reported that on one occasion he fainted twice. His tall spare frame, high forehead, and rather prematurely bald head, with merry eyes behind his large glasses, were the introduction to a personality which was widely loved and will be sadly missed. He was on a plane above most people, and Bristol is a poorer place for his departure.

The Annual Report of the Bristol Royal Hospital for 1943 pays him this tribute :—

Francis Wilfred Willway was appointed surgical Registrar in July, 1936, and temporary Honorary Assistant Surgeon in 1942. He possessed great organizing abilities and was made responsible for the reception of air-raid casualties, and for the admission and transfer of patients to E.M.S. Hospitals in this area. The arrangements made by him were carefully thought out and proved highly satisfactory.

He was Bristol's first Neuro-Surgeon and a leading authority in this country on the surgical treatment of mental disease. This was recognized by the Minister of Health who appointed him Deputy Regional Adviser on head injuries for the South West Region, shortly after the outbreak of war. The Royal College of Surgeons also recognized his abilities by electing him Hunterian Professor of Surgery in 1941. Mr. Willway was an outstanding personality who will be much missed.

A member of the permanent staff of the B.R.I. has written an appreciation from another angle, that of a non-medical fellow worker.

Of Francis Willway, Neuro-Surgeon, much has been written by appreciative colleagues; it is of Francis Willway the man and leader of men, that I would write.

Probably few B.R.I. Medical Officers had made themselves as completely *au fait* with the whole working of the House and its departments before the war. Early in 1940, before the blitzes had begun, there was much anxiety among the various departments as to what would happen if and when German bombers arrived. No official lead had been given and no adequate preparations made. Willway, with his finger on many strings, sensed the uneasiness, and one day, long to be remembered, called a staff meeting at which representatives of all departments were present—Residents, Sisters, and Nurses, Almoners, Medical Students, Works departments, Pharmacists, etc., with Mr. Chitty in the chair. There he tersely sketched the position, and appealed to us all to play our part. Enthusiastically supported, he submitted a scheme for night and day vigilance, fireguards, stretcher-bearers, and their duties, which was agreed to by all present. Willway that very night saw the President, and obtained the official blessing on the programme, which has worked continuously and harmoniously until this very week, when C.D. precautions were relaxed by the Home Secretary. Willway not only originated the service, but played his part in it with conspicuous *élan*. In the early days before the blitzes began, and also when they were at their height, he was always on the spot with a kindly word for every one, a never failing sense of humour, and the highest ideal of duty.

Few of us will forget him in that crowded O.P. department, sorting the cases, and getting them away without fuss to their wards. And the thing he started lived and grew, and from that day there was system and organization for the B.R.I. civil defence. To Francis Willway the B.R.I. owes it all. He was the Leader. "Ave atque vale."

---

As we go to press we learn with great regret of the death of Emeritus Professor E. W. Hey Groves, LL.D., D.Sc., M.S., F.R.C.S. A memoir of him will be published in our next issue. R.I.P.

